# The Effect of Silicon Grade and Electrode Architecture on the Performance of Advanced Anodes for Next Generation Lithium-Ion Cells

**DOI:** 10.3390/nano11123448

**Published:** 2021-12-20

**Authors:** Alexandra Meyer, Fabian Ball, Wilhelm Pfleging

**Affiliations:** Institute for Applied Materials-Applied Materials Physics (IAM-AWP), Karlsruhe Institute of Technology (KIT), Hermann-von-Helmholtz-Platz 1, 76344 Eggenstein-Leopoldshafen, Germany; Fabian.ball2@kit.edu (F.B.); Wilhelm.pfleging@kit.edu (W.P.)

**Keywords:** lithium-ion battery, electrode development, silicon anode, laser patterning, electrochemical impedance spectroscopy, galvanostatic characterization

## Abstract

To increase the specific capacity of anodes for lithium-ion cells, advanced active materials, such as silicon, can be utilized. Silicon has an order of magnitude higher specific capacity compared to the state-of-the-art anode material graphite; therefore, it is a promising candidate to achieve this target. In this study, different types of silicon nanopowders were introduced as active material for the manufacturing of composite silicon/graphite electrodes. The materials were selected from different suppliers providing different grades of purity and different grain sizes. The slurry preparation, including binder, additives, and active material, was established using a ball milling device and coating was performed via tape casting on a thin copper current collector foil. Composite electrodes with an areal capacity of approximately 1.70 mAh/cm² were deposited. Reference electrodes without silicon were prepared in the same manner, and they showed slightly lower areal capacities. High repetition rate, ultrafast laser ablation was applied to these high-power electrodes in order to introduce line structures with a periodicity of 200 µm. The electrochemical performance of the anodes was evaluated as rate capability and operational lifetime measurements including pouch cells with NMC 622 as counter electrodes. For the silicon/graphite composite electrodes with the best performance, up to 200 full cycles at a C-rate of 1C were achieved until end of life was reached at 80% relative capacity. Additionally, electrochemical impedance spectroscopies were conducted as a function of state of health to correlate the used silicon grade with solid electrolyte interface (SEI) formation and charge transfer resistance values.

## 1. Introduction

Due to the advancing electrification in mobility (xEV), for example in hybrid or electric cars (plug-in hybrid electric vehicle (PHEV), battery electric vehicle (BEV)) as well as e-bikes and e-scooters, it is necessary to reduce production costs and increase the production capacity of cells with high power and energy density. The goals of the European Union define a gravimetric energy density of 350 to 400 Wh/kg or a volumetric energy density of 750 Wh/L, which is to be achieved in cell generation 3b by approximately 2025 [1]. In particular, the increase in the driving range of BEVs and the fast-charging capability are current limiting factors for the successful integration of these vehicles into the automotive market. The cost per kilowatt-hour is also a decisive factor for the further establishment of BEVs.

To increase the power and energy density of Li-ion cells, advanced active materials can be utilized. On the cathode side, a decrease in cobalt content leads to the implementation of nickel-rich materials, such as NMC 622 or NMC 811, where the specific capacity is increased compared to the standard cathode material NMC 111 [2]. The use of novel anode materials with increased specific capacity, compared to the currently established material graphite with a theoretical specific capacity of 372 mAh/g (equivalent to LiC_6_), is of particular interest. In frame of the cell generation 3b concept, silicon (Si) is expected to be added to graphite composite anodes. As an anode material, silicon has a high theoretical capacity of 3579 mAh/g at room temperature (under formation of Li_15_Si_4_) and a potential against Li/Li+ of 0.4 V [3]. As silicon undergoes a large volume change of about 280% while cycling, the cycle stability and capacity retention are low [4]. To reduce the strain on the electrode material and therefore suppress the mechanical decomposition to a certain extent, a mixture of graphite and silicon can be utilized, which was already shown to be an effective way to implement silicon in anode materials [5]. When silicon nanoparticles (SiNP) with a diameter of <150 nm are applied, the crack formation, propagation, and eventual crumbling of the Si particles during Li insertion can be suppressed, as shown by Liu et al. [6]. Therefore, the ongoing formation of solid electrolyte interface (SEI) on the freshly exposed surface can be reduced and the consumption of electrolyte can be minimized. Several researchers have also utilized other approaches to incorporate silicon in electrodes by using specialized structures, for example with a yolk-shell structure [7,8], nanowires [9,10,11], hollow nanoparticles [12,13], or other more sophisticated setups [14] as well as by using different coatings [15]; however, the utilization of these materials and compositions in an industrial scale would be cost-intensive, which is why the use of “simple” nanoparticles is preferred.

Laser generated structures in the Si/graphite material are suitable to implement localized porosities, which reduces the residual stresses of the silicon-containing electrodes due to the volume change during electrochemical cycling and thus suppress the delamination of the electrode from the current collector and the resulting crumbling thereof, as well ensures good electrolyte wettability [16]. This approach was already utilized by Zheng et al. [5,17,18] for silicon-graphite anodes. Habedank et al. [19,20] structured graphite electrodes with blind holes, which also increased the specific discharge capacity. Smyrek et al. [21,22], Zhu et al. [23], and Song et al. [24] implemented line and grid structures for cathodes with various energy densities.

The present publication aims to emphasize the particular importance of selecting the appropriate starting materials, as well as to shed more light on the degradation mechanisms of silicon particles, to re-emphasize the advantages of laser patterning, and to highlight the next development steps that need to be taken for the successful implementation of silicon/graphite anodes in the commercial field. A comparative study of different types of silicon nanopowders was conducted. For this purpose, a silicon nanopowder content for the composite that is close to the current standard (about 3 wt.%) was chosen, i.e., 5 wt.%. The influence of the silicon on the mechanical integrity of the electrode was expected to remain low at this composition. For the same reason, thin electrodes were manufactured, which are also considered state-of-the-art.

## 2. Materials and Methods

### 2.1. Electrode Preparation

Silicon/graphite anodes were prepared via ball milling. Three different grades of commercially available SiNPs were used from three different material suppliers, namely manufacturer 1 (TSi), Nanografi Nanotechnology (NGP, Ankara, Turkey), and SkySpring Nanomaterials (SSN, Houston, TX, USA). The grades of silicon varied in purity and grain size, which will be shown later.

The chemical composition of the Si nanoparticles was measured with inductively coupled plasma optical emission spectroscopy (ICP-OES, iCAP 7600 DUO, Thermo-Fisher-Scientific, Waltham, MA, USA), carrier gas hot extraction (CGHE, G8 Galilei, Bruker AXS, Karlsruhe, Germany), and a carbon and sulfur analyzer (CS analyzer, TC600, LECO Instrumente, Mönchengladbach, Germany). The crystallinity was measured with X-ray diffraction (XRD, Empyrean, Malvern Panalytical, Malvern, UK) under an argon atmosphere in a non-ambient chamber (TTK 600, Malvern Panalytical, Malvern, UK) for diffraction angles 2Θ between 20° and 100°. Scanning electron microscope (SEM, Phenom Pro, Thermo Fischer Scientific, Waltham, MA, USA) images were prepared to characterize the shape and size of the SiNPs and their agglomerates.

The particle size of the coke-based graphite (SPGPT808, Targray, Kirkland, QC, Canada) was measured with laser scattering (LA-950, Horiba Europe, Oberursel, Germany), and the Brunauer–Emmet–Teller (BET) surface area was determined using nitrogen adsorption (Gemini VII 2390, Micromeritics, Norcross, GA, USA). The graphite particles were also examined using a SEM (Phenom Pro, Thermo Fischer Scientific, Waltham, MA, USA) to characterize their shape and size.

The spatial distribution of the graphite, silicon, and conductive carbon black at the surface of the electrode was examined with a scanning electron microscope (SEM, Zeiss Merlin, Carl Zeiss SMT AG, Oberkochen, Germany) and the distribution of the elements was measured with energy dispersive X-ray spectroscopy (EDX).

The compositions of the cathode and anode slurries are summarized in Table 1. For the anodes, a water based 2.5 wt.% sodium carboxymethyl cellulose (CMC, MTI Corporation, Richmond, CA, USA) solution was prepared with a vacuum mixer (MSK-SFM-7, MTI Corporation, Richmond, CA, USA) and subsequently stirred for 24 h with a magnetic stirrer. The graphite, conductive carbon black (CB, C-nergy Super C65, Imerys G&C, Paris, France), silicon nanopowder, and CMC solution were then premixed with a centrifugal mixer (Speedmixer DAC 150 SP, Hauschild, Hamm, Germany). The slurry was mixed with a ball mill (PULVERISETTE 7 premium line, Fritsch, Idar-Oberstein, Germany) to achieve a homogeneous distribution of the silicon nanoparticles. Styrene butadiene rubber solution (SBR, 50% solid content, MTI Corporation, Richmond, CA, USA) was added and slowly stirred into the mixture with a centrifugal mixer. The solid content of the anode slurry was 33.33%. The slurry was tape casted on a copper current collector (9 µm thickness) and dried at room temperature. The doctor blade distance was 150 µm. The anodes were subsequently calendered to reach a porosity (P) of 40%, calculated with the following equation:

$$P=\frac{l-w\left(\sum_{n}\frac{x_{n}}{d_{n}}\right)}{l}$$

With l being the thickness of the electrode, w the weight of the electrode per area, $x_{n}$ the mass fractions of the materials, and $d_{n}$ their corresponding densities (2.20 g/cm³ graphite; 2.33 g/cm³ silicon; 1.60 g/cm³ CMC; 1.05 g/cm³ SBR; 1.94 g/cm³ CB; 2.24 g/cm³ KS6L; 1.77 g/cm³ PVDF; and 4.63 g/cm³ NMC 622).

Single crystal NMC 622 (Targray, Kirkland, QC, Canada) cathodes were also prepared. PVDF (Solef 5130, Solvay GmbH, Hannover, Germany) and N-methyl-2-pyrrolidone solvent (NMP, BASF, Ludwigshafen, Germany) were premixed, then the NMC 622, conductive carbon black and conductive graphite were added. Additional NMP was used to adjust the viscosity. The solid content of the slurry was 66.67%. The cathode slurry was mixed in a centrifugal mixer and then tape casted on an aluminum current collector (20 µm thickness) with a doctor blade distance of 85 to 100 µm, depending on the required areal capacity to match the anodes with a cell balancing factor of 1.1 to 1.3. For the calculation of the cell balancing factor, a practical specific capacity of 172 mAh/g was used for the NMC 622, and 330 mAh/g was denoted for the graphite. In the applied voltage window between 3.0 and 4.2 V, a practical capacity of 2000 mAh/g was used for the silicon [4,25]. The NMC 622 electrodes were dried at 60 °C and subsequently calendered to reach a calculated porosity of 35%.

The dynamic viscosity of the anode slurry and the CMC solution was measured with a parallel plates viscometer (MCR72, Anton Paar, Graz, Austria) at 25 °C and a plate distance of 0.5 mm for shear rates from 1 to 100 s^−1^.

Laser patterning of the calendered anodes was performed with an ultrafast femtosecond (fs) fiber laser source (Tangerine, Amplitude Systèmes, Pessac, France) with a wavelength of 515 nm in ambient air. A repetition rate of 500 kHz and an average power of 1.5 W was applied to structure the electrodes. The scanning speed was kept constant at 500 mm/s. The process of laser ablation via scanning of the laser beam across the electrode surface was repeated several times until ablation up to the current collector was achieved. To assess the laser parameters and find the applicable number of scans, metallographic samples of the material were prepared and examined with a light microscope (Reicher-Jung MeF3, Leica Microsystems, Wetzlar, Germany). The reference electrodes remained unstructured. The laser structure consisted of a line pattern with a pitch of 200 µm. Electrodes for coin cells (CR2032) and pouch cells were laser cut. For the coin cells, circles with a diameter of 12 mm were cut. The pouch cells had a footprint area of 2492.3 mm².

### 2.2. Cell Assembly and Electrochemical Analysis

The electrodes for the coin cells were heated to 100 °C under vacuum for 24 h to remove excess moisture. The coin cells were assembled as half cells versus metallic lithium (Merck, Darmstadt, Germany) in an argon-filled glove box (argon 6.0, H_2_O < 0.1 ppm; O_2_ < 0.1 ppm). A polypropylene (PP) separator sheet (Celgard, Charlotte, NC, USA) with a thickness of 25 µm and a diameter of 19 mm was applied. As electrolyte, 120 µL of LP57 (EC:EMC 3:7, 1.3 M LiPF6) with 5 wt.% FEC (Gotion, Fremont, CA, USA) was utilized. It was previously shown by [26,27] that the addition of up to 5 wt.% FEC improves the performance of silicon/graphite electrodes. After stacking, the cells were sealed with an electric crimper (MSK-160D, MTI, Richmond, CA, USA) and stored at room temperature for 20 h to allow for a homogeneous wetting of the components with liquid electrolyte. The cells were then galvanostatically characterized using a battery cycler (Arbin Instruments, College Station, TX, USA). The voltage window was adjusted depending on the electrode composition: 0.01 to 1.5 V (anodes without silicon) or 0.06 to 1.2 V (anodes with silicon). The formation followed a constant current-constant voltage protocol. The cells that contained silicon were cycled with a C-rate of C/50 for one cycle, where the cut-off current in the constant voltage phase was set to C/100. After that, three more cycles with C/20 and a cut-off current at CV of C/50 followed. The formation of the other electrodes omitted the first formation cycle. After the formation step, the impedance of the cells was measured by electrochemical impedance spectroscopy (EIS) using a battery cycler (BCS810, Biologic, Seyssinet-Pariset, France) at 0% state of charge (SoC) between 30 Hz and 10 MHz with a voltage amplitude of 10 mV. Ten measurements per decade with logarithmic spacing and three measurements per frequency were carried out. Subsequently, the rate capability of the cells was measured with a constant current-constant voltage (CCCV) measurement protocol, the parameters of which are shown in Table 2. The currents were calculated based on the theoretical capacities of the active materials and the mass of the electrodes. Since the coin cells were cycled in a voltage window of 0.01 to 1.5 V/0.06 to 1.2 V, a practical specific capacity of 330 mAh/g was used for the graphite, and 3560 mAh/g for the silicon [4]. After the rate capability test, the cells were again cycled at C/5 for five cycles to characterize the capacity retention.

The electrodes that were to be assembled in pouch cells were dried inside the heated airlock of the glove box (M. Braun, Germany, argon 6.0, H_2_O < 0.1 ppm; O_2_ < 0.1 ppm) at 60 °C under vacuum for 24 h to remove the excess moisture. A polypropylene separator (25 µm thickness, Celgard, USA) with dimensions of 60 × 60 mm^2^ was used. After dry stacking, 3 mL LP57 with 5 wt.% FEC (as above) was added. The electrodes were soaked for 30 min, then the excess electrolyte was removed. After the cells were sealed, they were stored at room temperature for 20 h, until homogeneous wetting with the liquid electrolyte was achieved. They were cycled in a voltage window between 3 and 4.2 V at C/50 (CCCV, CV limit C/100) for one cycle, then degassed. For cells without silicon/graphite composite anodes, C/20 was chosen for the first formation cycle (CV limit C/50). The formation continued with three cycles of C/20 (CCCV, CV limit C/50). After a relaxation time of 24 h at 100% SoC, EIS measurements were performed between 10 Hz and 10 MHz with a voltage amplitude of 10 mV. Ten measurements per decade with logarithmic spacing and three measurements per frequency were carried out, followed by the symmetric rate capability test. The parameters of the rate capability test were identical to the parameters for the half cells, and are shown in Table 2. The cells were cycled in a voltage window between 3 and 4.2 V. After the rate capability test, the cells were again cycled at C/5 for five cycles to characterize the capacity retention. Another EIS measurement was performed at 100% SoC after the rate capability test; then, the long-term test with CCCV measuring protocol at a C-rate of 1C was performed. The C-rate was adjusted depending on the specific discharge capacity (SDC) of the cell at the fifth cycle of the rate capability test at 1C. After a minimum of 100 cycles, the EIS measurement was repeated.

## 3. Results and Discussion

In the following section, the results will be presented and discussed. First, the characterization of the raw materials and the manufactured electrodes will be shown. The galvanostatic characterization and the measurement of the electrochemical impedance spectra conclude the investigations.

### 3.1. Graphite Active Material

**Figure 1:**
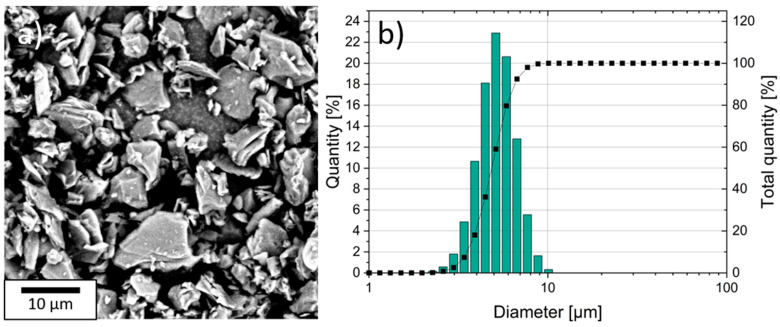
Scanning electrode microscope (SEM) image of the flake-like graphite (**a**) and particle size distribution (**b**).

The size and shape of graphite particles were examined using laser scattering and SEM. The results of the measurements are shown in Figure 1. The graphite particles had a flake-like morphology (Figure 1a). The median particle size (D50) of the graphite was 4.9 µm (D90 = 6.5 µm, D10 = 3.5 µm) and the particle size distribution had a Gaussian shape (Figure 1b). The measured BET surface area was 2.02 m²/g.

### 3.2. Silicon Nanopowders

**Figure A1:**
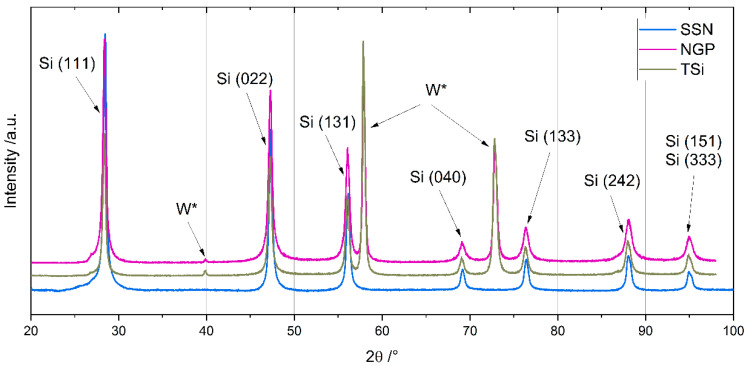
Reflections of the X-rays depending on the angle of incidence during XRD measurements of the Si nanoparticles; an offset was introduced for better visibility.

The results of the XRD measurements can be found in the Appendix A, see Figure A1. Reflections of the silicon were determined, as well as reflections from the tungsten substrate. The SSN silicon was measured with a different setup, where the tungsten crucible was omitted so that tungsten reflections could not be detected.

The Scherrer equation [28] was applied to determine the crystallite size of the silicon particles as follows:

$$L=\frac{K\cdot \lambda }{\Delta \left(2\theta \right)\cdot \cos\left(\theta_{0}\right)}$$

where L is the crystallite size, K is a form factor depending on the Miller indices of the analyzed reflections [29], and λ is the wavelength of the applied XRD source (Cu Kα with 1.5406 Å). The full width half maximum Δ(2θ) and Bragg angle $\theta_{0}$ were extracted from the XRD reflections (111), (022), (131), (040), (133), and (242). Applying the Scherrer equation, crystallite sizes of 17.7 ± 2.1 nm, 18.2 ± 0.9 nm, and 15.0 ± 2.9 nm were derived for TSi, SSN, and NGP silicon powder, respectively.

**Figure 2:**
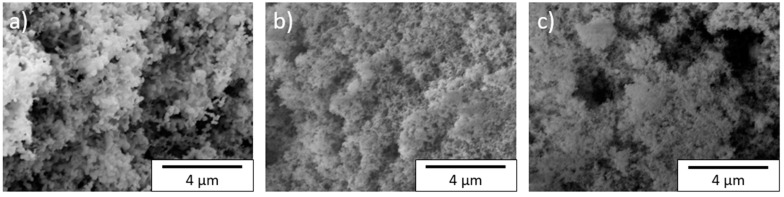
Scanning electrode microscope images of silicon particles at 10,000× magnification: (**a**) NGP, (**b**) TSi, and (**c**) SSN.

**Figure A2:**
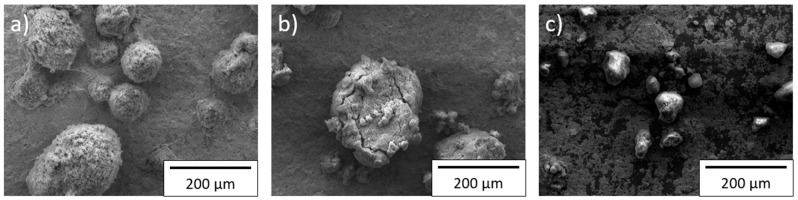
SEM image of the silicon particle agglomerates at 200× magnification for (**a**) NGP, (**b**) TSi, and (**c**) SSN.

Scanning electron microscopy images of the silicon particles and their agglomerates are shown in Figure A2a–c and Figure 2a–c. The agglomerates of the NGP silicon nanoparticles had a round appearance, while TSi agglomerates were more irregularly shaped and showed cracks. In comparison, SSN agglomerates were smaller and irregularly shaped, with a maximum diameter of approximately 50 µm, while the TSi and NGP silicon both showed agglomerates with a maximum diameter of approximately 200 µm. The SEM images showed large particles of NGP silicon, which can be seen in Figure 2a. The TSi and SSN SiNPs seemed to be in a similar size range, as can be seen in Figure 2b,c. Qualitatively, it was concluded that the particle size illustrated in the SEM images is larger than the crystallinity size evaluated from the XRD measurements. However, the evaluation of particle size with the Scherrer equation has been shown [30] to underestimate the actual size of particles when the particle size is larger than 50 nm, since particles might have more than one crystal boundary and might also show lattice strains, both of which broaden the XRD peaks.

The results of the chemical analyses of the silicon nanopowders are shown in Table 3. The SSN silicon powder showed the highest amount of impurities as well as the highest amount of oxygen. Traces of nitrogen, calcium, iron, and copper were also found. The other silicon nanopowders showed only traces of oxygen and carbon. Some types of impurities could not be assigned, leading to a sum of the mass percentages below 100%.

With the results of the XRD characterization, the SEM imaging, and the chemical characterization, three different grades of silicon nanopowders can be defined. TSi has a high purity and a smaller grain size, SSN has a lower purity and a smaller grain size, and NGP has a higher purity and a larger grain size.

### 3.3. Characterization of the Manufactured Electrodes

**Figure A3:**
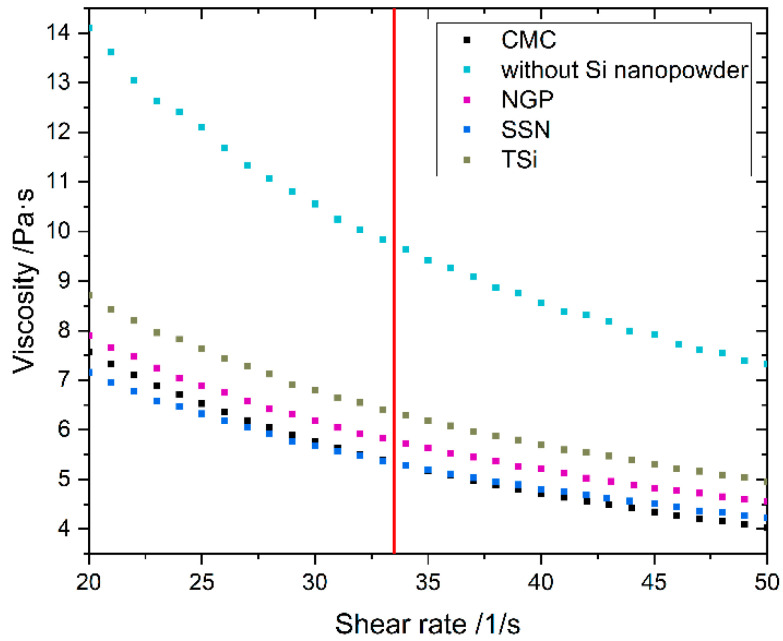
Viscosity of the slurries and the CMC solution for an application-oriented shear-rate range; the used shear-rate of 33.3 1/s is marked with a red line.

The viscosity of the anode slurries and the CMC solution were measured with a parallel plates viscometer. The results of the measurements are shown in Figure A3 for an application-oriented shear rate range. Since the electrodes were deposited with a velocity of 5 mm/s and a doctor blade gap of 150 µm, the material was sheared with a shear rate of 33.3 s^−1^, which is marked with a red line in Figure A3. The CMC solution, which is the main ingredient of the slurry and therefore has the highest impact on the viscosity, showed the lowest viscosity of 5.3 Pa s at 34 s^−1^. The anode slurry without silicon nanopowder showed the highest viscosity of 9.6 Pa s. The slurries with silicon addition showed a viscosity of 6.3 Pa s, 5.7 Pa s, and 5.3 Pa s for TSi, NGP, and SSN, respectively.

**Figure 3:**
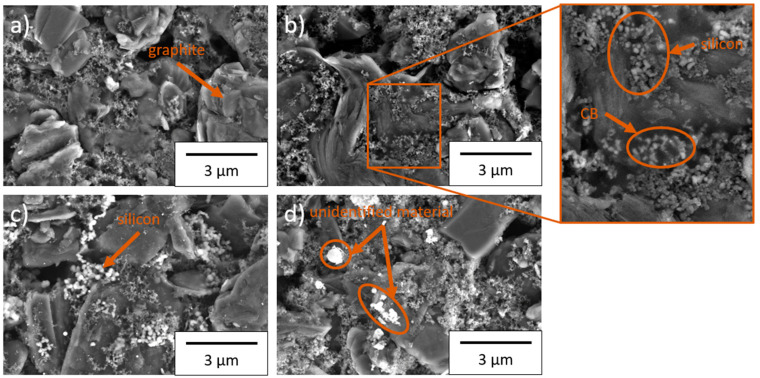
Scanning electron microscope images of the electrode surface at 10,000× magnification: (**a**) without silicon, (**b**) TSi, (**c**) NGP, and (**d**) SSN.

After the drying and calendaring of the deposited electrodes, scanning electron microscopy images of the electrodes were prepared (Figure 3). The graphite can be clearly identified and is marked in the figure. Due to the flake-like morphology of the particles, most have aligned horizontally (in parallel to the current collector), which impedes the lithium-ion insertion [31]. The silicon and conductive carbon black are distributed between the graphite and form electronic conductive paths. A small difference in size between the conductive carbon black and the TSi and SSN silicon can be identified by SEM (marked in Figure 3b detail view).

For the NGP silicon electrodes, the distinction between the silicon and the conductive carbon black is much clearer. The silicon is larger than the conductive carbon black and it appears lighter in the SEM image (Figure 3c). The SSN electrodes exhibited particles that appeared as white in the SEM images and therefore were not conductively connected to the rest of the material. They are marked in Figure 3d) and are of unclear origin. The electrodes were chemically characterized to confirm their composition. These analysis results are listed in Table 4. A high total percentage of all present elements was detected. The highest amount of Si was detected in the electrodes with NGP silicon with 5.3 wt.%, and the lowest in electrodes with SSN silicon with 4.5 wt.%.

The highest oxygen amount of 5.7 wt.% was measured in the electrodes with TSi silicon. The amount of carbon and sodium were very similar in all three types of Si/graphite electrodes, which means that the amount of binder, conductive carbon black, and graphite were almost constant. This leads to the conclusion that the highest amount of silicon oxides is present in the electrodes with TSi silicon. In this case, the ratio of oxygen to silicon was 2.11, while it was 1.34 for NGP electrodes and 1.39 for SSN electrodes. The preceding chemical analysis of the silicon nanopowders showed the highest amount of oxygen for the SSN nanoparticles (see Table 3). A rather constant amount of sulfur could be detected in all three samples. This may result from the synthetic graphite raw material, which is produced from petroleum coke that contains varying amounts of sulfur [32].

**Figure 4:**
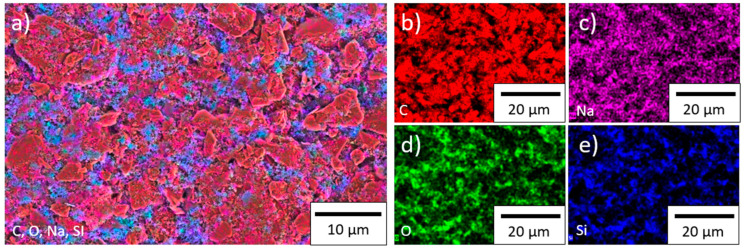
EDX measurements on the surface of a TSi electrode: (**a**) all detected elements, (**b**) carbon, (**c**) sodium, (**d**) oxygen, and (**e**) silicon.

**Figure A4:**
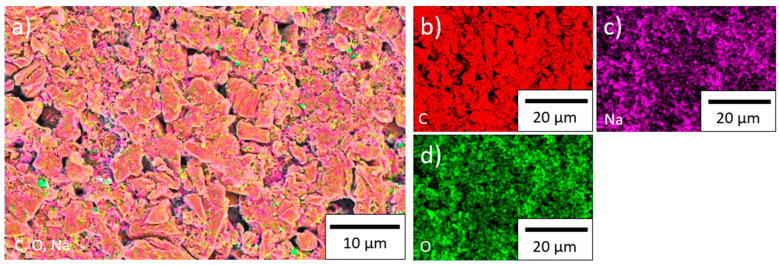
EDX measurements of the graphite electrode: (**a**) all detected elements, (**b**) carbon, (**c**) sodium, and (**d**) oxygen.

**Figure A5:**
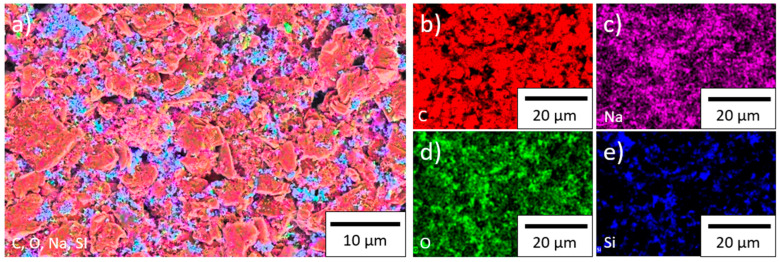
EDX measurements of the NGP electrode: (**a**) all detected elements, (**b**) carbon, (**c**) sodium, (**d**) oxygen, and (**e**) silicon.

**Figure A6:**
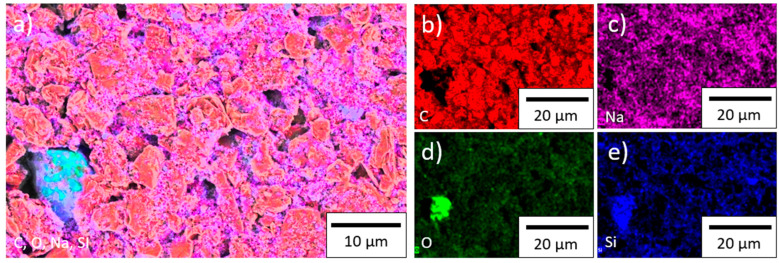
EDX measurements of the SSN electrode: (**a**) all detected elements, (**b**) carbon, (**c**) sodium, (**d**) oxygen, and (**e**) silicon.

EDX measurements of the surface of the electrodes were performed in order to characterize the spatial distribution of the components. The results for the electrodes with TSi silicon are shown in Figure 4, and the others can be found in the Appendix A (Figure A4, Figure A5 and Figure A6).

The silicon was mostly found in the space between the graphite particles. The binder contains sodium, which was mainly distributed between the graphite particles but could also be found as a layer on top of the graphite. Oxygen was detected between the graphite particles. The distribution of the oxygen and silicon overlapped in most cases, which supports the assumption that silicon and oxygen react during the slurry preparation, as suggested by [32], to form a thin oxide layer of SiO*_x_* (*x* ≤ 2) on the silicon nanoparticles; however, it is also in accordance with the previously measured presence of a natural oxide layer that is formed on the silicon raw material before processing (see Table 3). The EDX measurements for the electrode with SSN silicon showed an agglomeration of silicon (Figure A6), which represents a serious film defect that might be responsible for a fast electrochemical degradation during cycling. The other investigated electrodes showed a mostly homogeneous distribution of the elements.

**Figure 5:**
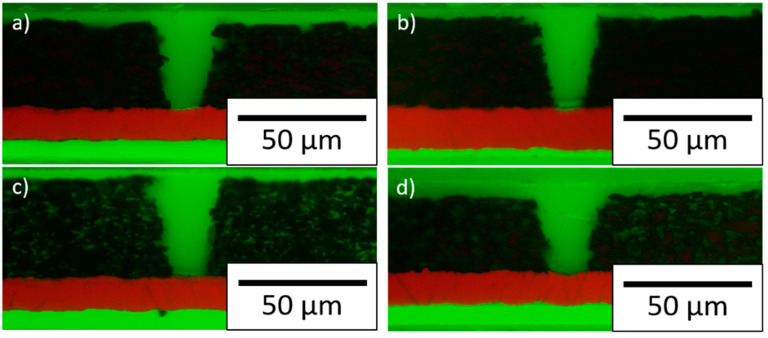
Cross sections of the structured electrodes: (**a**) graphite without silicon, (**b**) graphite with TSi, (**c**) graphite with NGP, and (**d**) graphite with SSN.

Cross sections of the structured electrodes are shown in Figure 5. The laser patterning process removed the material without damaging the current collector while implementing V-shaped lines. No debris formation on the electrode surface was detected. A line energy of 12 J/m (graphite, TSi, NGP) or 9 J/m (SSN) was applied for ablation. The dimensions of the structures are summarized in Table A1. A maximum aspect ratio of 2 could be achieved. At the top of the electrode, the lines had a mean width of 26.1 µm, while near the current collector they had a width of 11.8 µm.

After the drying, calendaring, and patterning of the deposited electrodes, the mean areal capacity was calculated (Table 5). A mass loss was observed due to the laser ablation of the material, which ranged from 6.4 to 14.9 wt.%, depending on the thickness of the electrodes and the shape of the manufactured grooves.

### 3.4. Galvanostatic Characterization

#### 3.4.1. Rate Capability Tests

**Figure 6:**
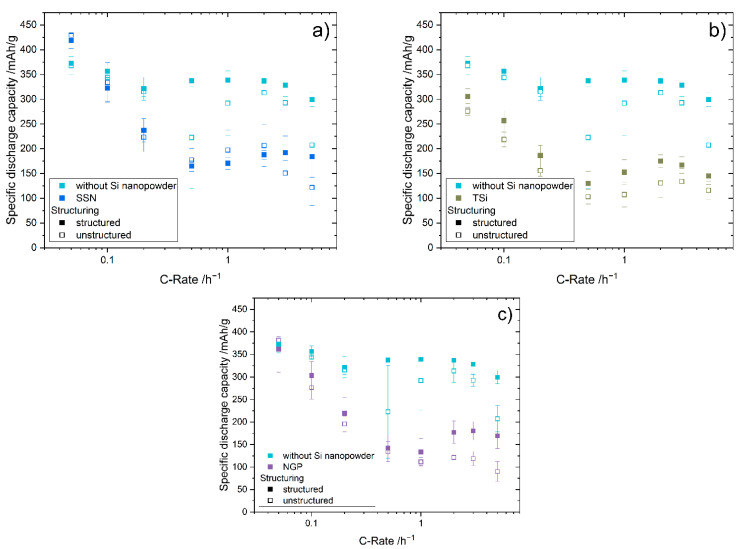
Mean specific discharge capacity of the half-cells depending on the C-rate of the structured and unstructured graphite electrodes without silicon and (**a**) electrodes with SSN SiNP, (**b**) electrodes with TSi SiNP, and (**c**) electrodes with NGP SiNP.

The mean specific discharge capacities (SDC) of the half cells for structured and unstructured silicon/graphite and graphite electrodes at C-rates between C/20 and 5C are shown in Figure 6. The mean SDC of all cells with silicon/graphite electrodes was lower than that for the reference cells with graphite electrode at C-rates larger than C/20. The values for the SDCs at C/20 are summarized in Table 6. Cells with unstructured electrodes with SSN silicon showed the highest SDC, while cells with unstructured electrodes with Tsi silicon showed the lowest SDC at C/20. Cells with SSN showed the highest SDC during formation compared to the cells with the other types of silicon. For SSN and NGP, the SDC during formation was lower for cells with the structured electrodes, while cells with graphite and Tsi showed a higher SDC. The cells with unstructured electrodes with SSN silicon showed a slightly higher mean SDC for C-rates of C/20, C/10, C/2, 1C, and 2C. At 3C and 5C, the SDC of cells with the structured SSN electrodes was 40 to 60 mAh/g higher than that of the unstructured electrode. For cells with the Tsi silicon electrodes, the benefit of the laser generated pattern was observed for each C-rate, and at 5C the SDC was increased by 30 mAh/g. Structured electrodes with NGP silicon provided an increased SDC for all C-rates except C/20, compared to the unstructured electrodes. At 5C, the SDC was increased by 80 mAh/g for the structured electrode. It has already been shown in several publications, for example [5,17,19,20,21,23], that the laser structuring of electrodes increases the SDC. In particular, the increased surface area provides new lithium-ion diffusion pathways, which improves the SDC at elevated C-rates. The reduction of mechanical stresses, particularly for the electrodes containing silicon, is another contributor for increasing SDCs, as was already established by Zheng et al. [18].

For most electrodes, SDC decreased with increasing C-rates until a minimum was reached at a C-rate of C/2 or 1C, after which the capacity increased again (Figure 6). The second maximum in capacity was reached at 2C or 3C, and at 3C or 5C the capacity decreased again. This behavior was observed for the unstructured SSN and NGP electrodes and both TSi electrodes, as well as the unstructured graphite electrodes. It was not as apparent for the structured SSN and NGP electrodes, where the capacity drop at 5C was not as pronounced as for the other electrodes. The cells with the structured graphite electrodes showed a very stable behavior, and the capacity did not show a strong increase or decrease depending on the C-rate, since they showed a different degradation behavior compared to the silicon/graphite electrodes. Additionally, the laser patterning enabled a high-power operation of these cells. The increase in capacity at higher C-rates might be explainable with an accelerating SEI formation, which binds high amounts of lithium. Hence, the measured capacity is increased, while the actual capacity decreases. At even higher C-rates (e.g., 5C), the negative impact of electrode degradation intensifies, and the measured capacity decreases again.

Since the standard deviations of the structured electrodes decreased compared to the unstructured electrodes in most cases, a more stable behavior during the rate capability tests was confirmed.

To measure the capacity retention, five additional cycles at a C-rate of C/5 were performed after the rate capability test. The results are listed in Table 6, as well as the capacity during the formation steps at C/20. The capacity retention increased for the structured electrodes for graphite and NGP, whereas it decreased slightly for SSN and TSi.

**Figure 7:**
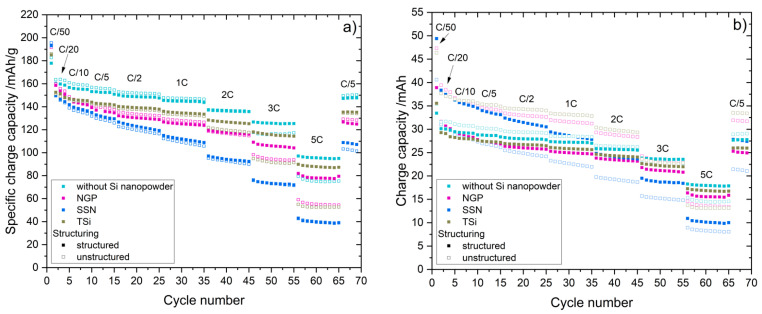
Rate capability tests of the pouch cells with unstructured and structured anodes. (**a**) Specific charge capacity (charge capacity normalized to the weight of the active material). (**b**) Charge capacity.

**Figure A7:**
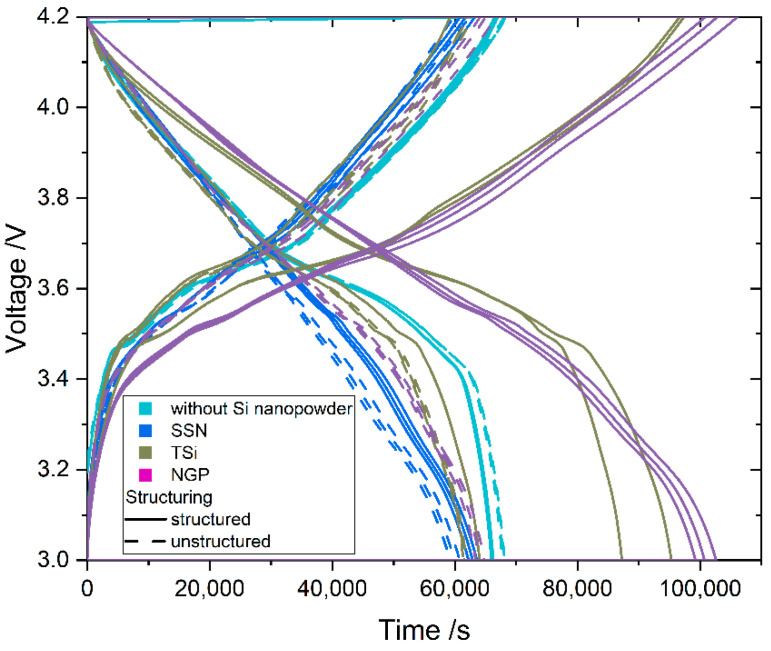
Voltage profiles of the pouch cells during formation at C/20.

The pouch cells were assembled and the cell balancing factor for each cell is summarized in Table A2. The aspired cell balancing factor of 1.2 was not achieved for all cells, particularly for the pouch cells with structured electrodes without silicon, which had an undersized anode with a cell balancing factor of 1.05. The charge capacities and specific charge capacities of selected pouch cells are shown in Figure 7, and the voltage profiles for the formation step at C/20 are shown in Figure A7.

**Figure A8:**
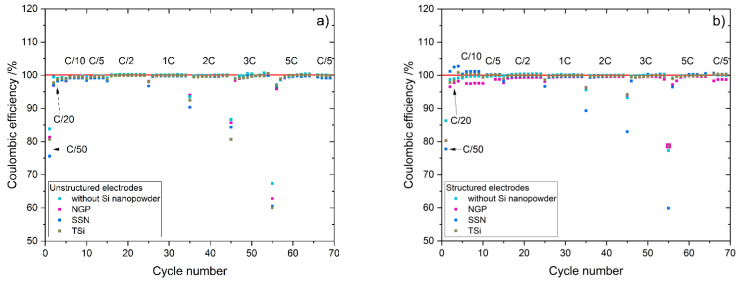
Coulombic efficiency of the pouch cells with (**a**) unstructured electrodes and (**b**) structured electrodes.

The highest specific capacities during the first formation cycle (C/50) were observed for the SSN electrodes at up to 196 mAh/g. The electrodes without silicon nanoparticles reached the lowest specific capacity of 178 mAh/g at the first formation cycle (C/20). After the first formation cycle, the capacity of all the cells decreased. At the lower C-rates (C/10 to 1C), the cells with unstructured and structured electrodes provided almost the same specific capacity, while at higher C-rates (2C to 5C), the impact of the laser patterning process could be observed, where the cells with structured electrodes had a higher specific capacity compared to those with unstructured ones. The cells with SSN silicon showed the highest drop in capacity with increasing C-rate, and at 5C the specific capacity of the SSN cells only reached approximately 40 mAh/g. The cells with unstructured NGP electrodes had a capacity at 5C ranging from 54 to 60 mAh/g, and the cells with the structured NGP electrodes still reached approximately 80 mAh/g. The cells with unstructured TSi electrodes reached 52 to 55 mAh/g, and the cells with structured TSi electrodes reached 87 to 89 mAh/g. At 5C, the highest specific capacity of 95 to 97 mAh/g was observed for cells with structured graphite electrodes, while the cells with unstructured graphite electrodes reached 75 to 79 mAh/g. The charge capacities of the pouch cells are shown in Figure 7b. The charge capacities of all cells with unstructured electrodes were higher than those with structured electrodes for C-rates up to 2C. At 3C and 5C, the capacity of the cells with structured electrodes was higher. The highest capacity of 49.4 mAh was reached at a C-rate of C/50 for a cell with SSN silicon; however, this cell also showed the highest drop in capacity. At 5C, the cell only had a remaining capacity of 10 mAh. For the pouch cells in general, the higher the capacity at low C-rates (i.e., C/50 and C/20), the lower the capacity at high C-rates (i.e., 3C and 5C).The Coulombic efficiency (CE) of the pouch cells is shown in Figure A8. When the C-rate changed, the CE dropped below 100% for the first cycle at a new C-rate and reached 100% again at the second or third cycle. The structuring reduced this CE drop. For example, at the first cycle of 5C, the unstructured TSi pouch cell had a CE of 60%, while the structured TSi pouch cell had a CE of 76%.

The capacity retention of the pouch cells is summarized in Table 7. As for the coin cells, the capacity retention increased when the electrode was laser structured. The increase was particularly high for electrodes with NGP silicon nanoparticles with 11.5%.

#### 3.4.2. Lifetime Tests

**Figure 8:**
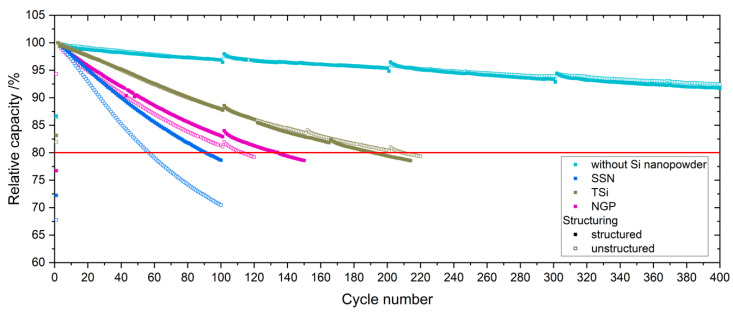
Relative capacity of the pouch cells during lifetime analysis at 1C/1C.

The relative capacity of the pouch cells with the best performance with structured and unstructured anodes during the lifetime tests at 1C (charge/discharge) is shown in Figure 8.

Only the first 400 cycles are shown for the electrodes without silicon. No major differences were observed between the relative capacities of the pouch cells with the structured and unstructured electrodes when only graphite was used as the anode material. When TSi silicon nanopowder was applied, the unstructured cells showed a slightly better performance with a longer lifetime and reached end of life (EoL) at 80% relative capacity after 202 ± 10 cycles, whereas the cells with structured electrodes only reached 182 ± 7 cycles. For NGP and SSN, the structuring of the anodes led to a higher relative capacity of the cells. The cells with unstructured anodes reached EoL after 51 ± 7 cycles and 105 ± 11 cycles for SSN and NGP, respectively. For the cells with structured anodes, the relative capacity dropped under 80% after 77 ± 10 cycles and 131 ± 3 cycles for SSN and NGP, respectively. This meant an increase of lifetime of 50.6% for SSN and 25.1% for NGP, whereas the electrodes with TSi showed a decrease in lifetime of 9.8%. The pouch cells with unstructured and structured reference electrodes did not reach end of life at this point. After 600 full cycles at 1C/1C, the pouch cells with unstructured and structured electrodes showed a relative capacity of 90% and 89%, respectively.

### 3.5. Electrochemical Impedance Spectroscopy

The impedance of the coin cells was analyzed with electrochemical impedance spectroscopy (EIS) after discharging to 0% SoC (0.06 V for half cells with silicon and 0.01 V for cells with pure graphite) and a relaxation time of 24 h. The pouch cells were analyzed with EIS at 100% SoC, and they were charged to 4.2 V and relaxed for 24 h. All spectra were verified with the Kramers–Kronig relation. The impedance was measured after the formation step, and EIS measurement followed the rate capability test and after EoL was reached for the pouch cells.

#### 3.5.1. EIS on Half Cells

**Figure 9:**
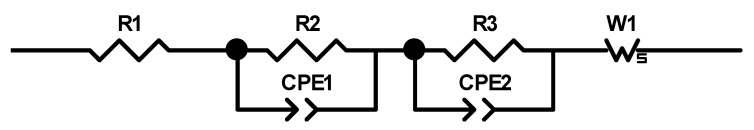
Equivalent circuit for the coin cells with ohmic resistances (R1, R2, R3), constant phase elements (CPE1, CPE2), and finite length Warburg element (W1).

The equivalent circuit used for the interpretation of the EIS data for the coin cells is shown in Figure 9. R_1_ accounts for the high frequency resistance of the electric contacts and the ionic pore resistance of the separator. The diffusion is represented by a finite length Warburg element W_1_. Since the measurements did not cover the inductive tail of the spectrum, an inductance was omitted. For the charge transfer processes and the polarization of the SEI, two resistances, R_2_ and R_3_, in parallel to constant phase elements (CPE) were chosen. Due to the very porous electrodes that were used in the cells, constant phase elements were chosen instead of capacitors [33].

**Figure 10:**
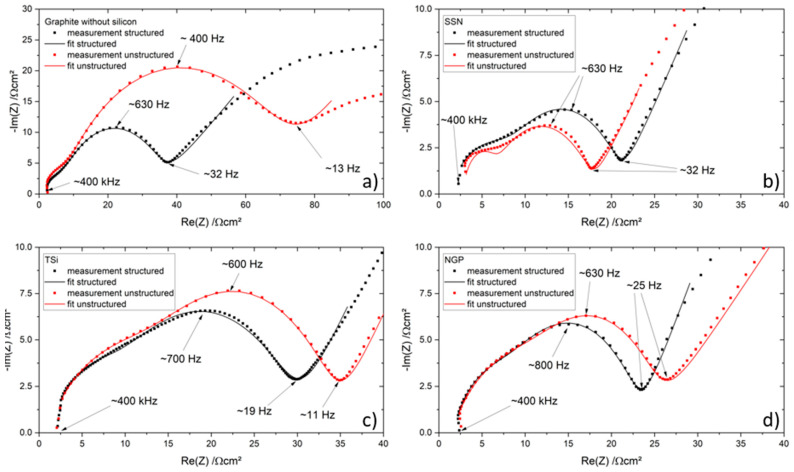
Electrochemical impedance spectra (Nyquist plots) of the coin cells with structured (black) and unstructured (red) electrodes and their corresponding fitting function: (**a**) pure graphite, (**b**) SSN, (**c**) TSi, and (**d**) NGP.

The results of the measurements as well as the corresponding fits can be found in Figure 10a–d, and the resistances are summarized in Table 8. The abscissa (R_1_) of the Nyquist plot is reached at an approximate frequency of 400 kHz for all coin cells. Since the same separator and coin cell case as well as the same measuring setup was used to characterize the cells, the ohmic resistance was almost constant for most cells and ranged from 2 Ω to 3 Ω, while the cell with an unstructured SSN electrode is the exception with a resistance R1 of 5 Ω. The first semicircles of the complex impedance at higher frequencies of the structured and unstructured electrodes showed the same size and curvature. Compared to the coin cells with pure graphite electrodes, all cells with silicon showed a larger semicircle at higher frequencies. This could be assigned to the different SEI composition and thickness. It was shown by Kalaga et al. [34] that the SEI on silicon particles is almost twice as thick as that on graphite particles. This increases the potential drop at the interfaces of electrode–SEI and SEI–electrolyte; therefore, a larger semicircle is observed.

The second semicircle at lower frequencies (approximately 400 Hz to 800 Hz) of the unstructured electrodes was larger compared to the semicircle of the structured electrode, with the exception of the SSN electrodes (Figure 10b). The frequency at which the maximum imaginary part of the impedance (Im(Z)) of the second semicircle was reached increased when the electrode is structured. Zheng et al. [5] also investigated the impedance of coin cells with structured and unstructured Si/C electrodes and found similar Nyquist plots. There, the smaller second semicircle of the laser structured electrode was assigned to a decreasing charge transfer resistance, due to improved lithium-ion diffusion kinetics and reduced compressive stresses. Habte et al. [35] investigated the effect of graphite electrode tortuosity on the impedance and observed a proportionality between them. Since laser structuring decreases the tortuosity by implementing additional lithium-ion diffusion pathways, the impedance is also reduced. However, the observed behavior of the SSN electrodes cannot be interpreted in the same way as suggested by the mentioned literature. The total capacity of the electrodes may be of importance for the direct comparison of different coin cells and their respective electrochemical impedance spectra. The measured SSN cell with an unstructured electrode had a total capacity of 2.06 mAh, while the SSN cell with a structured electrode had a capacity of 2.31 mAh. For all other setups, the total capacity of the cell with a structured electrode was smaller compared to the capacity of the cell with an unstructured electrode.

#### 3.5.2. EIS on Full Cells

The impedances were measured after the formation step, after the rate capability test, and after EoL was reached, or after 100 cycles at a C-rate of 1C. All spectra satisfied the Kramers–Kronig relation.

**Figure 11:**
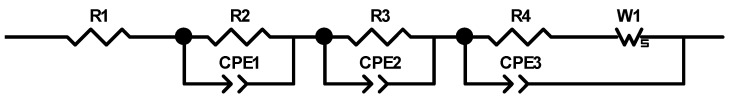
Equivalent circuit for the pouch cells with ohmic resistances (R1, R2, R3, R4), constant phase elements (CPE1, CPE2, CPE3), and finite length Warburg element (W1).

An equivalent circuit with three parallel R-CPE elements in series was used to account for the charge transfer processes of the anode and cathode, and the polarization of the interface between the electrode and the electrolyte due to the SEI [36], which is the most appropriate model for this application [37]. The high frequency resistance of the electric contacts and the ionic pore resistance of the separator was modeled by a resistance, R_1_. Since the maximal applied frequencies were not sufficient to cover the inductive tail of the spectrum, an inductance was omitted. The diffusion of the lithium ions in the bulk material is represented by a finite length Warburg element W_1_, which is incorporated in the third R-CPE element. The equivalent circuit is shown in Figure 11.

**Figure 12:**
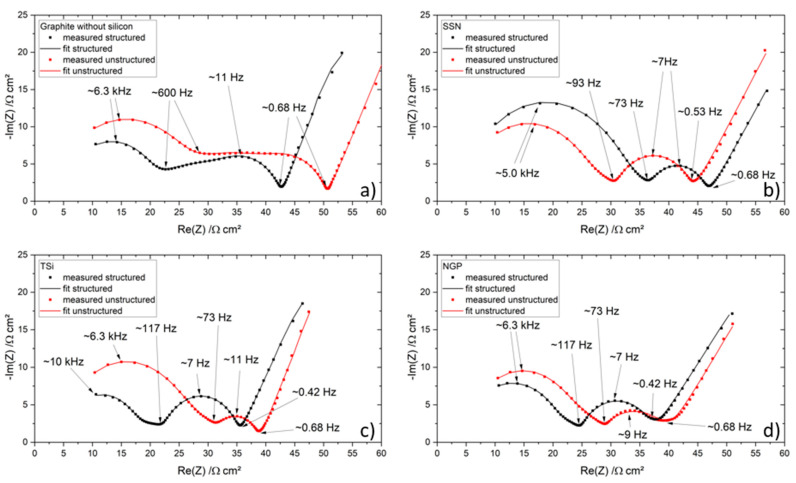
Electrochemical impedance spectra of the pouch cells after the formation step and their respective fit: (**a**) graphite, (**b**) SSN, (**c**) TSi, and (**d**) NGP anode.

The Nyquist plots of the measurements taken after the formation step, as well as the results of the modelling, are shown in Figure 12. The values of the resistances of all measurements are summarized in Table A3. The calculated values for the ohmic resistance R_1_ were very similar for all cells, in a range of 0.12 Ω to 0.23 Ω. When the cell aged, no prominent increase or decrease was observed for R_1_, since the electric contacts of the measurement setup stayed constant. The first semicircle of the pouch cells with the graphite electrodes (unstructured and structured, Figure 12a) did not show a clear separation until the second or third semicircle, which led to a plateau-shaped graph. For the cell with a structured electrode, the first and second semicircles were smaller compared to the cell with an unstructured electrode.

The Nyquist plots of the impedances for the pouch cells with silicon/graphite electrodes showed a more pronounced separation of the first and second semicircle from the third one. Most of the cells with silicon-containing anodes could also be fitted with very good agreement to only two R-CPE elements, which would complicate the interpretation and comparison of the data. In the Nyquist plot, the first semicircle is therefore interpreted as two merged semicircles, when applicable. The pouch cells with TSi and NGP SiNPs behaved in a similar manner regarding the size and relation of the Nyquist plots, while the pouch cells containing SSN behaved inversely (see Figure 12b–d). The transition between the first/second semicircle and the third semicircle occurred for all cells with silicon/graphite anodes in a frequency range of 73 Hz to 117 Hz.

The transition between the mid-frequency range and the low frequency range was identified between 0.42 Hz and 0.68 Hz for all pouch cells. The total resistance of the cells with unstructured graphite, TSi, and NGP silicon was larger compared to the resistance of their structured counterpart, while the SSN pouch cells showed a larger total resistance for the structured pouch cell.

It seems to be evident that the laser structuring of the electrodes led to a diminishment of the two semicircles at higher frequencies (except for the SSN pouch cells), which is mostly associated with the interface between surface films (e.g., SEI) and the liquid electrolyte [38,39]. For the cells containing silicon, with the exception of the SSN pouch cells, the laser patterning process led to a widening of the third semicircle, which is mostly associated with the charge transfer and the double layer capacitance [38,39,40], while the semicircle at lower frequencies did not change its appearance for the graphite pouch cells regardless of the laser patterning.

**Figure 13:**
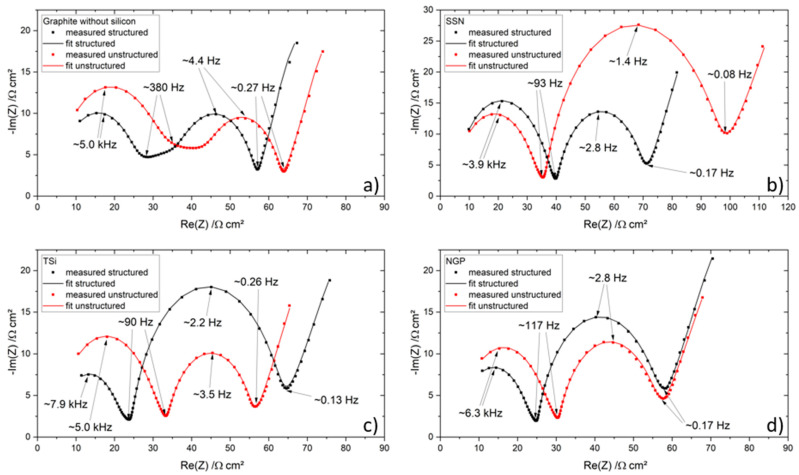
Electrochemical impedance spectra of the pouch cells after the rate capability test and their respective fit: (**a**) graphite, (**b**) SSN, (**c**) TSi, and (**d**) NGP anode.

After the rate capability test, the pouch cells were again characterized with EIS, and the results are shown in Figure 13. A shift to higher resistances and a widening of the semicircles compared to the results of the EIS measurements after formation due to ongoing degradation processes and hence increasing internal resistance and capacitance of the cell were demonstrated, which was also previously shown in [41,42]. The pouch cells with graphite still showed three separate semicircles, while the first and second semicircle of the unstructured pouch cell were larger, and the third semicircle was just as big as the semicircles of the structured cells (Figure 13a). In total, the resistance of the unstructured pouch cell was larger, as it was also shown after the formation.

For the pouch cells containing silicon (Figure 13b–d), no separation between the first and second or second and third semicircle was observed. For the SSN pouch cell, the total resistance of the unstructured cell was larger compared to the structured cell. For the TSi pouch cells, the resistance of the cell with the structured electrode was bigger. The NGP pouch cells showed a similar behavior to the TSi pouch cells; however, the total resistance of the structured and unstructured cells was comparable.

**Figure 14:**
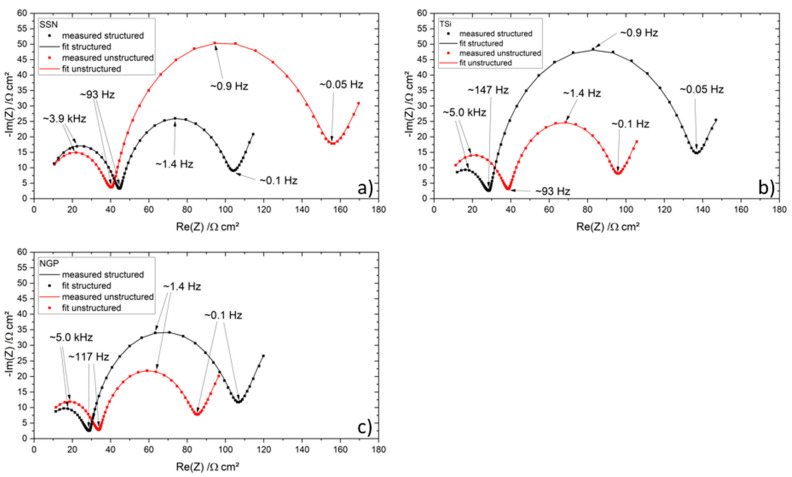
Electrochemical impedance spectra of the pouch cells after EoL and their respective fit: (**a**) SSN, (**b**) TSi, and (**c**) NGP anode.

Figure 14 shows the Nyquist plots of the pouch cells containing silicon after EoL was reached. The pouch cells with a pure graphite anode did not reach EoL in the frame of this study and were therefore excluded. Compared to The Nyquist plots of the measurements taken after the formation step EIS data after the formation and the rate capability test, a shift to larger resistances and an ongoing widening of the semicircles was again observed, as was also reported by [41,42]. The pouch cells with SSN silicon showed a slightly larger first and significantly smaller second semicircle for the structured cell (Figure 14a) while the NGP and TSi pouch cells showed the opposite behavior (Figure 14b,c) as was also observed after the formation (Figure 12c,d) as well as after the rate capability test (Figure 13c,d). The total resistance of the structured TSi pouch cell was increased by about 150% compared to the total resistance of the unstructured cell, while the structured NGP pouch cell showed an increased resistance of 125% compared to the unstructured cell. For the SSN pouch cells, the unstructured cell still showed a higher total resistance than the structured cell. When the impedances of the cells with ongoing ageing are compared, a shift to lower frequencies for the maximum of the two semicircles can be observed, as was also reported in [41], where it was associated with a decelerating charge transfer process and an increasing cell polarization by the SEI.

The noticeable contrary behavior of the SSN pouch cells might be explained by the total capacity. The cell with a structured electrode showed a larger capacity for all C-rates compared to the cell with an unstructured electrode (see Figure 7), while the specific capacities were comparable for both setups. For all other cell compositions, the capacity of the unstructured cell was larger for C-rates up to 3C. A direct inclusion of the capacity values in the impedance is not expedient. Additionally, an overall inhomogeneous distribution of the silicon due to an insufficient breaking of the silicon agglomerates during mixing was observed for the SSN electrodes, and was shown by the EDX measurements (Figure A6) as well as the presence of impurities in the silicon raw material detected by the chemical analysis (see Table 3). The EIS of the SSN pouch cells after EoL was reached was measured after 100 cycles at 1C; however the state of health of the cell with an unstructured electrode was lower than that of the structured cell (Figure 8), which may have impacted the results shown in Figure 14.

In conclusion, the first/second semicircle of the Nyquist plots (except for the SSN pouch cells) was minimized when the electrodes were laser structured. This behavior was observed over the entire lifetime of the cells. The size of the semicircle at lower frequencies was increased when the electrodes were structured (with the exception of the SSN pouch cells). This behavior was more pronounced when silicon was used.

The semicircles at high frequencies are mostly associated with the lithium-ion migration through the SEI [39]. With the laser patterning, a more homogenous development of the SEI was enabled, since the wetting with liquid electrolyte is improved due to the capillary effect. This leads to a reduction of the semicircles at higher frequencies for laser structured electrodes.

The semicircle at lower frequencies is mostly associated with the double layer capacitance, the charge transfer, and the inter-particle electron transfer [38,39,40]. It was shown for the coin cells that the structured cells showed a reduction of the semicircle at lower frequencies, which could be associated with a decreasing charge transfer resistance due to improved lithium-ion diffusion kinetics and reduced compressive stresses. For the structured pouch cells, the semicircle at lower frequencies was larger. The most significant difference between the pouch cells and the coin cells is the amount of available lithium. In the coin cell, a large surplus of lithium is present. Therefore, the loss of lithium due to the formation of SEI can be compensated. Further studies with full cells where prelithiated anodes or overlithiated cathodes are employed might document this behavior more precisely. The voltage window that is applied may also play a significant role. The reduction of compressive stresses plays a minor role for the thin film electrodes in the pouch cells, since the strict compression by the springs and the rigid components of the coin cell are not present here. The overall increased reactance of the silicon-containing pouch cells may be associated with the increased inter-particle electron transfer [40], since the total amount of particles is increased when a nanomaterial is used.

## 4. Conclusions and Outlook

Three different SiNPs were used to manufacture silicon/graphite composite electrodes for lithium-ion cells. Additionally, reference cells with graphite were prepared. First, the raw materials were thoroughly characterized. After the tape casting, drying, and calendaring of the electrodes, a high repetition rate, ultrafast-laser source was utilized to implement line structures in the electrode. The chemical composition of the electrode and the spatial distribution of the materials within the electrode were examined. The cells were assembled in coin cells vs. lithium and pouch cells vs. NMC 622 electrodes, and were subsequently galvanostatically characterized to measure their rate capability and lifetime. Additionally, the electrochemical impedance data of selected cells were measured by EIS.

It was confirmed that the laser patterning process can vastly improve the lifetime and the fast charging and discharging capability of silicon/graphite and graphite electrodes assembled in coin and pouch cells, even when the areal capacity, and therefore thickness, is low and when suboptimal raw materials and compositions are used.

The graphite reference cells showed the most stable electrochemical performance and a higher specific discharge capacity when assembled in coin cells compared to cells with electrodes containing silicon. This is not surprising since the study was carried out with thin film electrodes, which are normally used in high power applications, and excellent performance of graphite electrodes is to be expected. Nevertheless, a low areal capacity was selected in order to identify the most promising silicon nanopowder to be adapted in future silicon/graphite electrodes with high mass loading and optimized electrode architecture.

The three different silicon grades showed an immense range of performances, but electrodes containing TSi showed a stable behavior and high specific discharge capacities during the rate capability test when assembled in coin cells. When silicon/graphite electrodes are assembled in pouch cells, the difference between electrodes with and without silicon dwindle, and the cells with structured electrodes containing TSi SiNP achieve specific discharge capacities at high C-rates almost comparable to the graphite electrodes. During lifetime analysis, the electrodes containing TSi SiNP achieved up to 200 full cycles at a C-rate of 1C until EoL was reached at 80% of initial capacity. Therefore, TSi SiNP is the most promising candidate for further development.

The inherent degradation mechanisms of silicon cannot be overcome by the laser patterning, as was shown with the measurement of the electrochemical impedance spectra. Even directly after the formation, the influence of the different SEI chemistries of graphite and silicon becomes apparent, as the reactance and resistance are increased when silicon is used. With diminishing state of health, the disparity between the graphite and silicon/graphite electrodes increases. The direct comparison of EIS data of different cells with varying capacities and states of health should be treated with caution, as it was proven that the capacity in particular has a yet to be defined impact on the impedance.

The European Union targets for gravimetric and volumetric energy densities [1] could not be met with the materials and methods used; however, future research efforts will continue to pursue this goal. The use of different graphite materials will be revised, since some publications [31,43,44] showed that the use of chamfered graphite particles could increase the capacity of the cells. It was shown with EDX measurements and SEM imaging that the spatial distribution and size of the components play a role in the successful application of electrodes in Li-ion cells; therefore, the slurry preparation will be revised, and the lateral distribution of the components will be characterized with laser induced breakdown spectroscopy. It was also shown [45,46,47,48] that the use of different binders (Na-CMC, SBR, PVDF) with different molecular weights and degrees of substitution plays a major role; hence, the amount and type of binder will also be amended. An overlithiation of the cathode or prelithiation of the anode may also increase the capacity of cells with silicon/graphite composite electrodes, which was shown in [49,50], and will also be examined in future studies. It was shown by Berckmans et al. [51] that a pressurization of the cells during cycling may also be favorable. To further understand the mechanisms in the cells, EIS will also be performed at different SoCs.

## Figures and Tables

**Table 1:** Composition of the electrode slurries.

Anode Material	Mass Fraction /wt.%	Cathode Material	Mass Fraction /wt.%
Graphite	80	NMC 622	92
Si-NP	5	graphite KS6L	2
C65	5	C65	3
CMC	5	PVDF	3
SBR	5		

**Table 2:** Parameters of constant current-constant voltage (CCCV) measurements.

**C-rate**	C/10	C/5	C/2	1C	2C	3C	5C
**Limit (CV)**	C/20	C/10	C/10	C/10	C/10	C/10	C/10
**Repetitions**	5	5	10	10	10	10	10

**Table 3:** Results of chemical analyses for the silicon nanomaterials used, measured by inductively coupled plasma optical emission spectroscopy (ICP-OES), carrier gas hot extraction (CGHE), and carbon and sulfur (CS) analyzer (LoQ: limit of quantification).

		NGP		TSi		SSN	
	Unit	$\bar{x}$	±	$\bar{x}$	±	$\bar{x}$	±
Si	wt.%	98.2	4.9	98.2	4.9	86.7	4.3
O	wt.%	0.445	0.035	0.866	0.068	11.5	0.9
C	wt.%	0.0037	0.0009	0.0059	0.0030	0.0373	0.0037
N	wt.%	<LoQ	-	<LoQ	-	0.715	0.123
Ca	wt.%	<LoQ	-	<LoQ	-	0.0007	0.0002
Fe	wt.%	<LoQ	-	<LoQ	-	0.0010	0.0002
Cu	wt.%	<LoQ	-	<LoQ	-	0.0029	0.0002
Sum	wt.%	98.65		99.07		98.96	

Measurement uncertainty was calculated based on DIN ISO 11352 with coverage factor k = 2.

**Table 4:** Chemical composition of the manufactured electrodes measured by ICP-OES and CGHE.

		NGP		SSN		TSi	
	Unit	$\bar{x}$	±	$\bar{x}$	±	$\bar{x}$	±
C	wt.%	87.02	7.40	88.38	7.51	87.30	7.42
Si	wt.%	5.29	0.26	4.46	0.22	4.74	0.24
O	wt.%	4.04	0.32	3.55	0.28	5.71	0.45
Na	wt.%	0.46	0.01	0.46	0.01	0.44	0.01
S	wt.%	0.03	0.01	0.03	0.01	0.02	0.01
Sum	wt.%	96.83		96.88		98.21	

Measurement uncertainty was calculated based on DIN ISO 11352 with coverage factor k = 2.

**Table 5:** Areal capacity and mass loss of the electrodes after manufacturing and subsequent laser structuring.

Electrode	Areal Capacity, Unstructured mAh/cm²	Areal Capacity, Structured mAh/cm²	Mass Loss wt.%
Graphite without Si	1.33 ± 0.03	1.15 ± 0.01	13.5
TSi	1.61 ± 0.02	1.37 ± 0.02	14.9
SSN	1.56 ± 0.15	1.46 ± 0.15	6.4
NGP	1.64 ± 0.04	1.40 ± 0.02	14.6

**Table 6:** Specific discharge capacity of the coin cells at formation and capacity retention at C/5 calculated with the first five cycles at C/5 and the last five cycles at C/5.

Electrode	Formation Unstructured	Formation Structured	Capacity Retention Unstructured	Capacity Retention Structured
	/mAh g^−1^	/mAh g^−1^	/%	/%
Graphite	368 ± 18	373 ± 20	100	107
SSN	429 ± 27	419 ± 13	116	114
TSi	276 ± 8	306 ± 15	121	112
NGP	381 ± 8	361 ± 51	104	107

**Table 7:** Capacity retention (CR) of the pouch cells with unstructured and structured anodes at C/5, calculated with the first five cycles at C/5 and the last five cycles at C/5.

Material	CR Unstructured/%	CR Structured/%	CR Increase/%
Without Si	86.2	88.8	3.0
NGP	79.9	89.1	11.5
SSN	67.3	72.0	7.0
TSi	84.3	87.6	3.9

**Table 8:** Resistances of the coin cells, extracted from Figure 10.

Material	R_1_/Ω	R_2_/Ω	R_3_/Ω
Graphite unstructured	2.500	7.708	113.100
Graphite structured	2.419	9.632	46.980
SSN unstructured	5.000	5.548	20.460
SSN structured	2.124	13.200	21.280
TSi unstructured	3.388	9.985	48.740
TSi structured	3.331	7.046	41.870
NGP unstructured	2.575	20.430	19.880
NGP structured	3.035	12.920	22.700

## Data Availability

Data sharing is not applicable to this article.

## Appendix A

**Table A1:** Measurements of the structures that were generated by a laser beam.

Material	Width Top w_t_/µm	Width Middle w_m_/µm	Depth d/µm	Aspect Ratio d/w_m_
Graphite	25.85	19.33	34.40	1.78
SSN	28.14	17.71	30.32	1.71
TSi	22.70	16.51	35.03	2.12
NGP	27.83	18.60	38.804	2.09

**Table A2:** Cell balancing factor of each pouch cell.

Patterning	Without Silicon			TSi			SSN			NGP		
Unstructured	1.16	1.12	1.20	1.26	1.25	1.11	1.28	1.30	1.27	1.20	1.19	1.12
Structured	1.05	1.05	1.05	1.21	1.22	1.20	1.15	1.21	1.05	1.20	1.18	1.20

**Table A3:** Resistances of the pouch cells after formation and the rate capability test, and at EoL.

	R_1_/Ω	R_2_/Ω	R_3_/Ω	R_4_/Ω
Graphite without silicon unstructured				
Post-formation	0.126	0.296	0.886	0.714
Post-RCT	0.163	0.632	0.979	0.763
Graphite without silicon structured				
Post-formation	0.191	0.305	0.587	0.626
Post-RCT	0.189	0.665	0.756	0.661
SSN unstructured				
Post-formation	0.182	0.497	0.808	0.261
Post-RCT	0.180	2.353	0.984	0.301
EoL	0.193	4.320	1.122	0.401
SSN structured				
Post-formation	0.188	0.304	1.008	0.366
Post-RCT	0.206	1.183	1.140	0.271
EoL	0.229	0.279	1.333	2.269
TSi unstructured				
Post-formation	0.192	0.274	0.693	0.397
Post-RCT	0.203	0.865	0.862	0.298
EoL	0.213	2.128	1.067	0.341
TSi structured				
Post-formation	0.214	0.467	0.409	0.312
Post-RCT	0.226	1.510	0.485	0.284
EoL	0.231	4.162	0.590	0.395
NGP unstructured				
Post-formation	0.212	0.450	0.686	0.230
Post-RCT	0.206	1.015	0.822	0.198
EoL	0.217	1.907	0.873	0.312
NGP structured				
Post-formation	0.168	0.479	0.678	0.140
Post-RCT	0.190	1.242	0.697	0.122
EoL	0.218	2.940	0.658	0.327
